# Supplementary material for: PI 3‐kinase delta enhances axonal PIP 3 to support axon regeneration in the adult CNS
Source: EMBO Mol Med. 2020 Jun 17;12(8):e11674. doi: 10.15252/emmm.201911674 (PMC7411663; doi:10.15252/emmm.201911674)
Supplement: Supplementary file 1 — Expanded View Figures PDF [file EMMM-12-e11674-s001.pdf]

Expanded View Figures

Figure EV1. Gene expression profile of p110 isoforms in the nervous system from previously published RNAseq datasets.

Panels A to C show data from Tedeschi *et al* (2016), Panels D and E from Koseki *et al* (2017), and Panels F to M from Brain-RNAseq databases (http://www.brainrnaseq.org/), Zhang *et al* (2014) and (2016).

A Normalised mean expression values of p110 genes in adult mouse DRG neurons isolated after sciatic nerve lesion compared to a sham control.
B Normalised mean expression values of p110 genes in cultured mouse DRG neurons 6-, 12, 24- and 36 h post-plating (representing the shift from arborising to elongating axon growth).
C Normalised mean expression values of p110 genes in cultured mouse DRG neurons from at embryonic days 12.5 and 17.5.
D Relative abundance of p110 mRNA levels in cortical neurons cultured from E18 rat embryos at increasing periods of time *in vitro*.
E Relative abundance of p110 mRNA levels in cortical neurons cultured from E18 rat embryos at increasing periods of time *in vitro*, also showing expression levels other regeneration-associated genes.
F–M Relative abundance (FPKM) of p110 genes in various mouse and human brain cell types (astrocytes, neurons oligodendrocyte precursor cells, newly formed oligodendrocytes, myelinating oligodendrocytes, microglia/macrophages and endothelial cells). Each replicate consists of pooled cortices from 3 to 12 mice. For human samples, n = 6–12 for each cell type. Data are shown as the mean ± SEM. See Zhang *et al* (2014) and (2016) for full details.

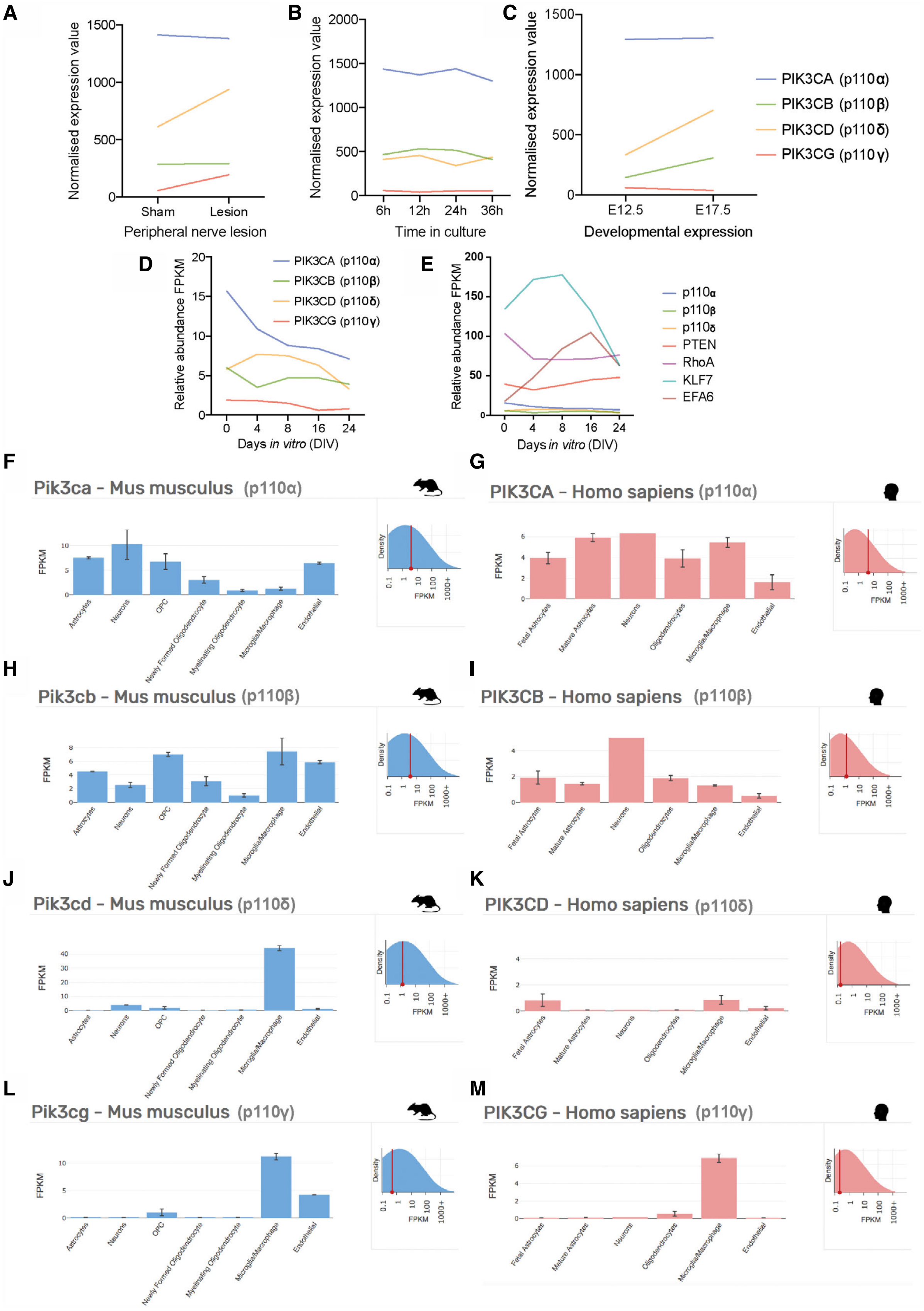

Figure EV1.

© 2020 The Authors

Figure EV2. Validation of a protocol for detecting PIP_3 in neuronal membranes.

A Time-lapse images (single confocal section imaged by spinning disc microscopy) of a DRG growth cone cultured from adult AKT-PH-GFP mice. Arrows point to hotspots/regions of increased fluorescence indicative of AKT-PH recruitment. See also associated Movie EV1.

B Time-lapse images (single confocal section imaged by spinning disc microscopy) of a DRG growth cone cultured from adult AKT-PH-GFP mice, stained with cell-mask orange (magenta colour) to detect the membrane. Arrows indicate a dynamic region of AKT-PH-GFP recruitment that does not label for membrane aggregation. See also associated Movie EV2.

C Adult DRG growth cone cultured from AKT-PH-GFP mice, fixed for PIP_3 immobilisation (see methods section) and labelled with an antibody to PIP_3 (magenta). Arrows and dotted circles indicate colocalisation.

D Non-neuronal cell from a dissociated DRG culture from AKT-PH-GFP mice, fixed for PIP_3 immobilisation (see methods section), and labelled with an antibody to PIP_3 (magenta). Arrows indicate colocalisation. Inset highlights colocalisation at a large region of AKT-PH-GFP recruitment.

E N1E neuroblastoma cells stimulated with insulin and labelled for PIP_3 in the presence (lower panels) or absence (upper panels) of the pan-PI3K inhibitor GDC-0941. Cells were co-labelled for F-actin to show cell density (right four panels). Left four panels are heatmap to show fluorescence intensity. Middle four panels are grey scale images of PIP_3 fluorescence.

F Quantification of PIP_3 in N1E neuroblastoma cells stimulated with insulin and in the presence or absence of the pan-PI3K inhibitor GDC-0941. Insulin leads to an increase in PIP_3, which is not detected in the presence of GDC-0941. n = 15 fields of view from 3 experiments. Data are shown as the mean ± SEM. *P*-values indicate significance measured by ANOVA with Tukey's *post-hoc* analysis.

G Quantification of F-actin in N1E neuroblastoma cells stimulated with insulin and labelled for PIP_3 in the presence or absence of the pan-PI3K inhibitor GDC-0941. n = 15 fields of view from 3 experiments. Data are shown as the mean ± SEM. *P*-values indicate significance measured by ANOVA with Tukey's *post-hoc* analysis.

© 2020 The Authors

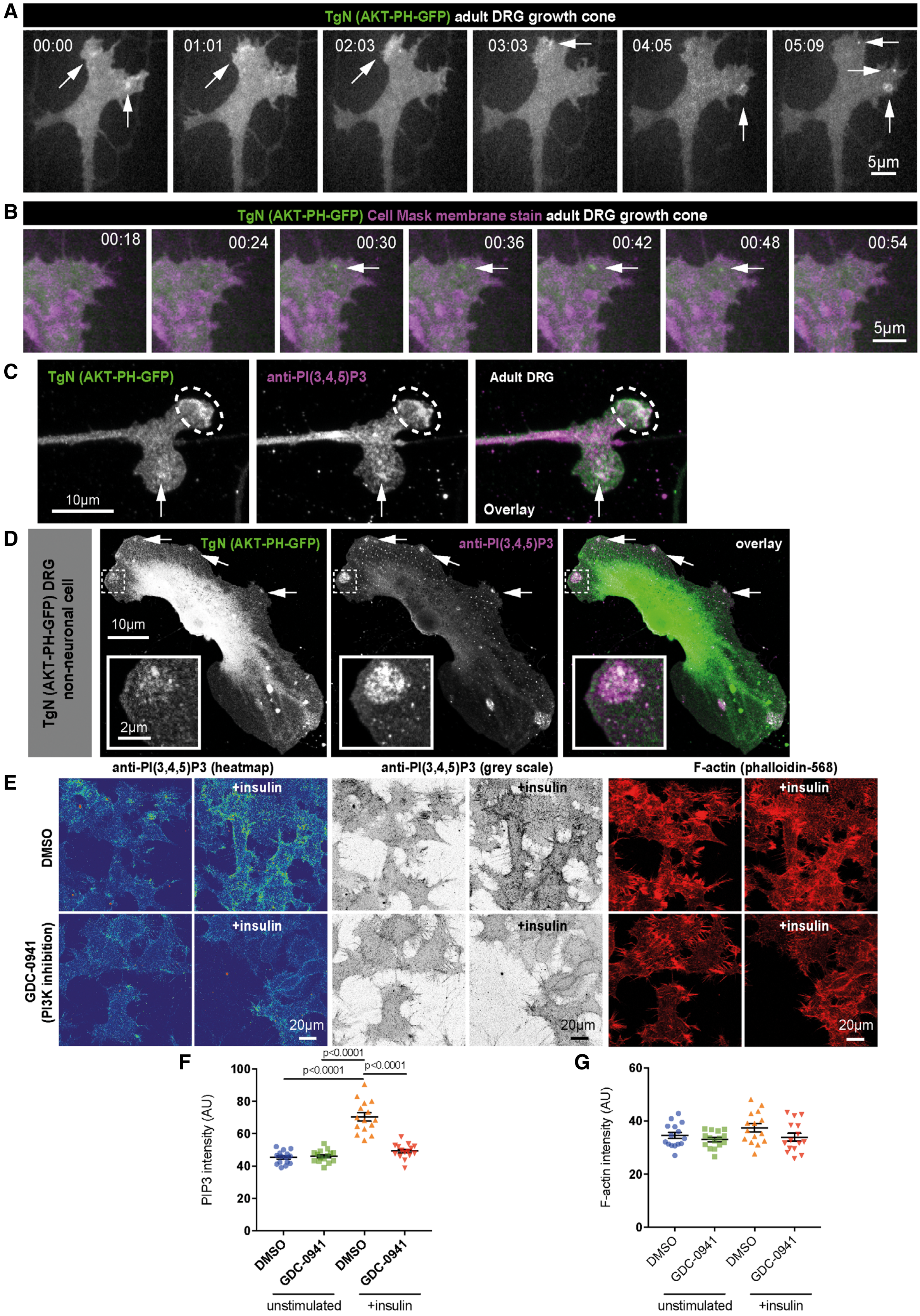

Figure EV2.

© 2020 The Authors

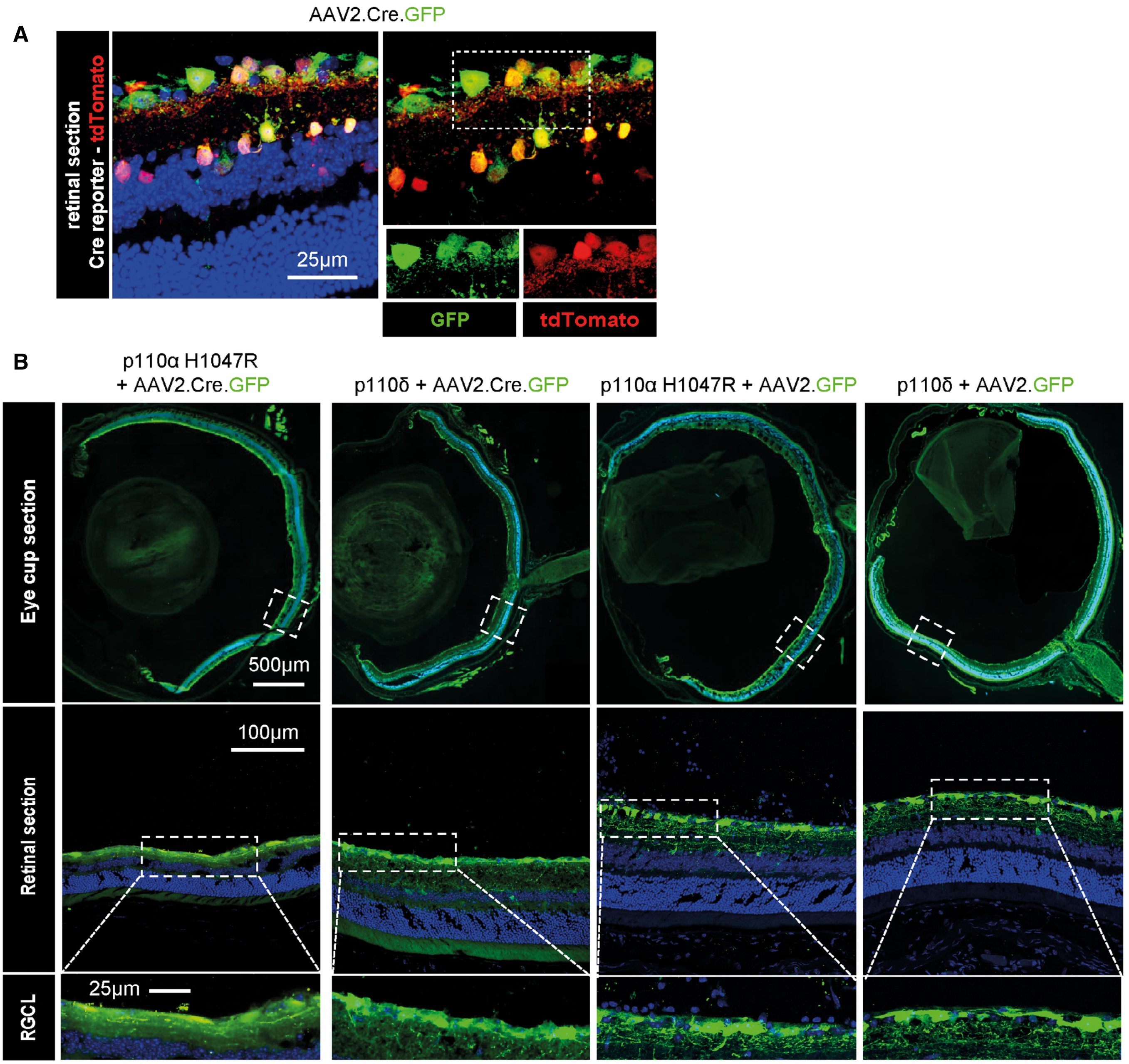

Figure EV3. Validation of recombinant adeno-associated viral vectors and targeting of mouse RGC neurons.

A Representative image showing expression of AAV2.Cre.GFP and co-localisation with TdTomato in Rosa26 Cre-reporter mice 2 weeks after injection of virus. Inset images show individual colours at the same scale as the full image. Blue colour is DAPI nuclear staining.

B Representative images showing expression of AAV2 vectors in eye cup and retinal sections in the four treatment groups as indicated, 2 weeks after injection of virus. Images highlight expression in RGC layer (lower panels). Blue colour is DAPI nuclear staining.

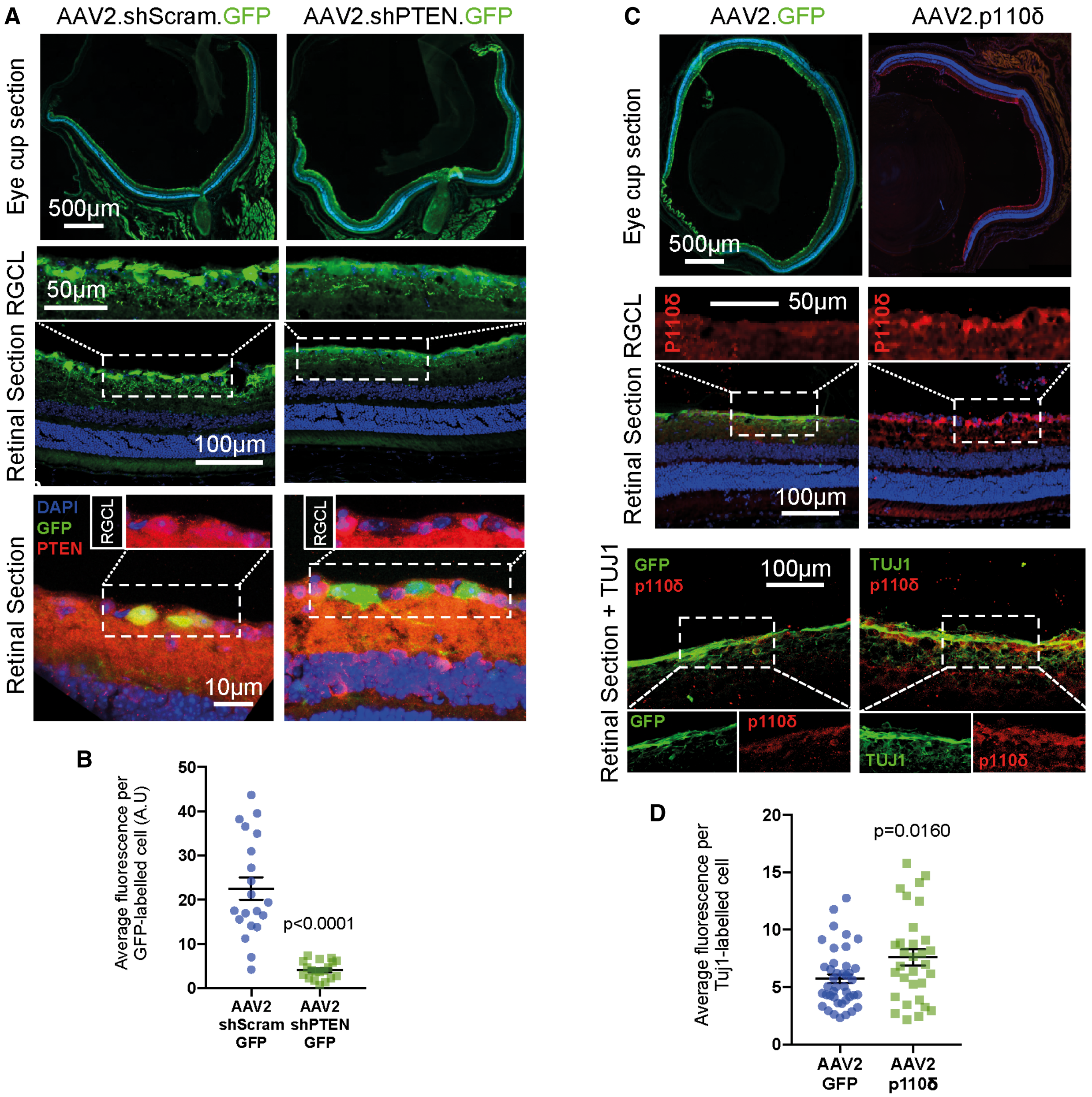

Figure EV4. Confirmation of AAV-mediated PTEN reduction and AAV transduction of p110δ in the mouse retina.

A Representative confocal images showing expression of AAV2.shScram.GFP (control) and AAV2.shPTEN.GFP in eye cup and retinal cross-sections. Lower panels show a reduction in PTEN immunofluorescence in GFP-positive cells in the RGC layer 2 weeks after injection with AAV2.shPTEN.GFP. Lower panel inset images show only PTEN immunofluorescence at the same scale as the full image. Blue colour is DAPI to indicate nuclei.

B Quantification of the PTEN immunofluorescence in transduced RGCs. n = 20 for each condition. Data are shown as the mean ± SEM. *P*-values indicate statistical significance as measured by two-tailed Student's *t*-test.

C Representative images showing expression of AAV2.GFP and AAV2.p110δ in eye cup and retinal sections 2 weeks after AAV injection. Retinae from eyes injected with AAV2.p110δ (right-hand panels) were labelled for TUJ1 (green) and p110δ (red). Blue colour is DAPI to indicate nuclei. Lower panel inset images show individual colours at the same scale as the full image.

D Quantification of the p110δ immunofluorescence in RGCs transduced with AAV2.p110δ. n = 41 for AAV2.GFP, 31 for AAV2.p110δ. Data are shown as the mean ± SEM. *P*-values indicate statistical significance as measured by two-tailed Student's *t*-test.

 © 2020 The Authors
